# Multiple step saccades are generated by internal real-time saccadic error correction

**DOI:** 10.3389/fnins.2023.1112655

**Published:** 2023-02-28

**Authors:** Wenbo Ma, Mingsha Zhang

**Affiliations:** State Key Laboratory of Cognitive Neuroscience and Learning and IDG/McGovern Institute for Brain Research, Division of Psychology, Beijing Normal University, Beijing, China

**Keywords:** reactive saccades, anti-saccade, memory-guided saccade, internal model, multiple step saccades

## Abstract

**Objectives:**

Multiple step saccades (MSSs) are an atypical form of saccade that consists of a series of small-amplitude saccades. It has been argued that the mechanism for generating MSS is due to the automatic saccadic plan. This argument was based on the observation that trials with MSS had shorter saccadic latency than trials without MSS in the reactive saccades. However, the validity of this argument has never been verified by other saccadic tasks. Alternatively, we and other researchers have speculated that the function of MSS is the same as that of the corrective saccade (CS), i.e., to correct saccadic errors. Thus, we propose that the function of the MSS is also to rectify saccadic errors and generated by forward internal models. The objective of the present study is to examine whether the automatic theory is universally applicable for the generation of MSSs in various saccadic tasks and to seek other possible mechanisms, such as error correction by forward internal models.

**Methods:**

Fifty young healthy subjects (YHSs) and fifty elderly healthy subjects (EHSs) were recruited in the present study. The task paradigms were prosaccade (PS), anti-saccade (AS) and memory-guided saccade (MGS) tasks.

**Results:**

Saccadic latency in trials with MSS was shorter than without MSS in the PS task but similar in the AS and MGS tasks. The intersaccadic intervals (ISI) were similar among the three tasks in both YHSs and EHSs.

**Conclusion:**

Our results indicate that the automatic theory is not a universal mechanism. Instead, the forward internal model for saccadic error correction might be an important mechanism.

## 1. Introduction

Saccades are rapid eye movements that direct the fovea onto various interested objects. A typical saccade comprises of a primary saccade which could cover all or most distance from the fixation point to the target, which might be followed by a saccade with small-amplitude corrective saccade (CS) if required. Nevertheless, eyes do not always jump with the typical style but occasionally with a series of at least two smaller amplitude (hypometric) saccades, i.e., multiple step saccades (MSSs) (Troost et al., 1974). It has been reported that the incidence of MSS is sex independent (Ma and Zhang, 2022) but age-related with an asymmetric “U” shape (Van Donkelaar et al., 2007; Litvinova et al., 2011; Ma and Zhang, 2022). This “U” shape consists of two processes, i.e., the developmental process from young childhood to adulthood and the natural degeneration process from adulthood to elderly (Van Donkelaar et al., 2007; Litvinova et al., 2011; Ma and Zhang, 2022). Moreover, the incidence of MSS significantly increases in patients with neurodegenerative diseases, particularly in Parkinson’s disease (PD) patients (Jones and DeJong, 1971; Corin et al., 1972; Troost et al., 1974; Teräväinen and Calne, 1980; White et al., 1983; Hotson et al., 1986; Lueck et al., 1990, 1992; Van Gisbergen et al., 1992; Kimmig et al., 2002). Therefore, it has been argued that MSS could be a behavioral biomarker for the early diagnosis of PD (Blekher et al., 2009; Ma et al., 2022).

Despite the advanced knowledge of the relationship between the rate of MSS and age, sex and neurodegenerative diseases, the neural mechanisms underlying MSS generation are less studied. To the best of our knowledge, it has only been argued in one study that the occurrence of MSS was due to a automatic saccadic plan (Van Donkelaar et al., 2007). This argument was based on the observation that trials with MSS had shorter saccadic latency than trials without MSS in a visually guided reactive saccade task (Van Donkelaar et al., 2007). However, the validity of this argument has never been verified by other saccadic tasks, e.g., voluntary saccade tasks such as anti-saccades and memory guided saccades. Alternatively, since the function of MSS has been considered to be the same as CS, i.e., to rectify the spatial errors of saccades (Becker and Fuchs, 1969; Oliva, 2001), saccadic error correction by a forward internal model, i.e., predicting future sensory inputs from the combination of the current state of the saccadic system and an efference copy of the current saccadic command (Wolpert et al., 2006). Might be a possible mechanism for MSS generation (Kawato, 1999; Mehta and Schaal, 2002). The rationale of the forward internal model requires two internally generated signals, i.e., the desired (intentional) and actual (executional) eye displacement signals, to generate an error signal and trigger MSS (Robinson, 1973; Kawato, 1999; Mehta and Schaal, 2002; Wolpert et al., 2006).

To verify the validity of automatic theory and examine the internal model hypothesis, we compared the saccadic latency between trials with and without MSS in three tasks (verifying the automatic hypothesis) and compared the intersaccadic intervals (ISI) among three tasks as well as between MSSs and CSs in the prosaccade task.

## 2. Materials and methods

### 2.1. Participants

Fifty young healthy subjects (YHSs) and fifty elderly healthy subjects (EHSs) were recruited in the present study. The demographics of the participants in the present study are shown in Table 1. We recruited the subjects from the college and residential community. We ascertained the sample size by G-power software (Faul et al., 2007), with an effect size of 0.45, α of 0.05, β of 0.1 and power of 1-β of 0.9. In addition, each participant has completed the Folsteinmini-mental state examination (MMSE) with a minimum score of 27 to exclude the effect of cognitive impairments. All participants had normal or corrected-to-normal vision. All participants were informed about the requirement to perform each task and provided written consent to take part in the study. However, they did not know anything about the purpose of the experiments.

**TABLE 1:** Demographic and clinical characteristics of the subjects.

	YHS	EHS
N (male/female)	50 (29/21)	50 (14/36)
Age in years^a^	23.08 ± 3.39	65 ± 7.72
MMSE^a^	29.21 ± 0.95	28.48 ± 1.05

^a^Mean ± SD.

### 2.2. Experimental design

We employed three saccade tasks, i.e., the pro-saccade task (PS), anti-saccade task (AS) and memory-guided saccade task (MGS), in the present study. Each task was run in separate blocks, and each block consisted of 40 trials. In addition, the orders of different blocks were counterbalanced from PS, AS, and MGS among participants. Each participant spent approximately 15 min to complete the experiment.

#### 2.2.1. Pro-saccade task

**FIGURE 1:**
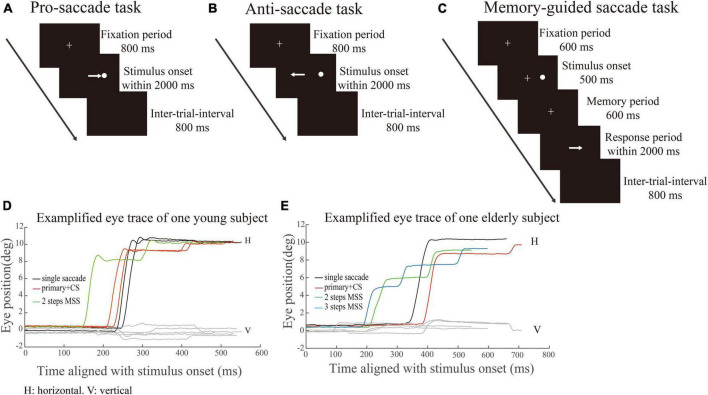
Schematic illustration of saccadic tasks. White crosses and circles represent fixation points and targets, respectively. The white arrow represents the required saccade. **(A–C)** Represents pro-saccade task (PS), anti-saccade task (AS) and memory-guided saccade task (MGS), respectively. **(D,E)** Represents exemplified eye position of one YHS and EHS in PS, respectively. *X*-axis indicates the time (ms) aligned with the stimulus onset. *Y*-axis denotes the eye position. Black, red, green, and blue traces indicate single saccades, one primary saccade followed by a CS and saccades with 2 or 3 MSSs, respectively.

Figure 1A Each trial began with a white cross appearing at the center of screen for 800 ms. The participant needed to stare at the white cross within 800 ms (check window 4° in radius) and remained fixation for 300 ms, and then the white cross disappeared. Simultaneously, a white dot (saccadic target) randomly appeared at one of four peripheral locations with 10° eccentricity. The participants were required to make a saccade toward the target as quickly and precisely as possible. The target disappeared only after the eye got into and held in the check window (radius: 4°) for 300 ms. The size of the fixation points and target were 1° in length or diameter, respectively. The inter-trial interval was 800 ms with an interposed blank screen.

#### 2.2.2. Anti-saccade task

Figure 1B The AS task consisted of the same sequence of events as in the PS task, except that the participants were asked to make a saccade toward the opposite direction (mirror location) of the target.

#### 2.2.3. Memory-guided saccade task

Figure 1C Each trial began with a white cross (fixation point) appearing at the center of the screen. Participants needed to look at the fixation point as soon as possible (within 800 ms) after its appearance and remained fixated (check window 4° in radius) as long as it was on. After 600 ms, a white dot (target) randomly appeared at one of four peripheral locations (right, left, up and down; eccentricity of 10°) for 500 ms. Participants were required to keep the central fixation for an additional 600 ms (memory period) and remember the target location. Participants were required to make a saccade toward the remembered target location only until the fixation point disappeared. The size of the fixation points and target in MGS were the same as those in PS. The inter-trial interval was 800 ms with an interposed blank screen.

### 2.3. Data acquisition

We employed a head-restrained infrared video-based eye tracker (EM-2000R, Jasmine Science and Technology Ltd., Beijing, China; Eye Link 1000 desktop mount, SR Research, Ltd., Ontario, Canada). The sample rate of the eye tracker was 1 kHz. Participants were seated in a dark room 57 cm away from the monitor (XL2720-B; 27-inch; refresh rate: 100 Hz resolution: 1920 × 1080). We calibrated the eye tracking system before each experiment for each participant by having the participants fixating at nine locations (composed of a 3 × 3 rectangle). The luminance of background and visual stimuli were 0.08 and 23.9 cd/m^2^, respectively. In the present study, we employed MATLAB (R2009b; MathWorks, Natick, MA, USA) with Psychtoolbox (PTB-3) running on a Windows system PC (HP) to control stimuli presentation and behavioral data collection.

### 2.4. Quantitative measures of MSS

We employed the similar criteria as being reported by our laboratory for the quantitative measurements of MSS and CS in the PS task (Ma et al., 2022). In PS, MSS was defined as if a saccadic event met any one of the following criteria: 1. The saccadic number within the saccadic event is ≥ 3; 2. The saccadic number within the saccadic event is two, and the amplitude of the first responsive saccade is < 7°; 3. The saccadic number within a saccadic event is two, the amplitude of the first responsive saccade is ≥ 7°, and the amplitude of the secondary saccade is ≥ the threshold, i.e., mean + 1.5*std of the secondary saccadic amplitudes. The directions of all mentioned saccades are the same. As for the definition of MSS in AS and MGS, we considered all small amplitude saccades (> 1°) as MSS. The percentage of the distance to the target was covered by the single saccade and MSS was 97.6 and 63.5% in PS, 86.9 and 73.8% in AS, 88.4 and 66.8% in MGS for YHS; 85.5 and 68.8% in PS, 84 and 62.4% in AS, 75.3 and 65.9% in MGS for EHS. The exemplified eye positions were shown in Figures 1D, E for a YHS and EHS in PS, respectively. Black, red, green, and blue traces indicate single saccades, one primary saccade followed by a CS and saccades with 2 or 3 MSSs, respectively. Meanwhile, to compare the saccadic latencies of trials with and without MSS, we defined trials without MSS in PS, AS and MGS separately. The trials without MSS in PS were single saccades or one primary saccade followed by one small CS, whereas the trials without MSS in AS and MGS tasks were single saccades. The latencies of saccades in the same direction were compared between trials with and without MSS. To ensure a sufficient number of trials during this analysis, we set 4 as the minimum trial number for trials with or without MSS. The aim of this analysis is to compare the saccadic latency between trials with and without MSS. Therefore, we set the minimum number of trials with MSS not less than 4, to ensure there are enough trials in each group, particularly in group with MSS, for making comparison. We also made the same analysis with the minimum number of trials with MSS being 5, 6, and the results were similar. We could not set the minimum number of trials with MSS any bigger, because the incidence of MSS was lower as we show in below. The average number of trials with/without MSS was 4/34, 4/33, and 8/28 in PS, AS, and MGS for YHS; 7/29, 8/24, and 6/25 in PS, AS, and MGS for EHS. Thus, the number of subjects used in this analysis was 10 and 25 in PS, 38 and 26 in AS, and 20 and 19 in MGS for YHS and EHS, respectively. The ISI was the time from the end of the preceding saccade to the start of the present saccade.

In addition, since the total number of trials in each session was 40, to ensure that there was a sufficient number of correct trials for data analysis, the incidences of MSS and CS were calculated when the correct rate of a session was ≥ 50%. The correct rate was defined as the number of correct/required saccades divided by the total number of trials. We firstly calculated the correct rate in each task for individual subject. Then, calculated the incidence of MSS only when the correct rate was ≥ 50%. We also calculated the mean correct rates in the PS, AS, and MGS tasks, and resulted with 96.91, 77.64, and 57.05% for the YHS group and 93.62, 54.64, and 44.88% for the EHS group, respectively. To assure our analysis is valid, i.e., with a sufficient number of trials, we pooled the four directions together and calculate the incidences and ISIs of MSS.

### 2.5. Statistical analysis

The Kruskal–Wallis test was applied to determine the significant difference of the incidence of MSS and the ISI among three tasks and two groups of subjects, respectively. This was corrected by the Bonferroni correction with α being set to 0.05. If there were significant differences among PS, AS, and MGS tasks and YHS and EHS, a *post hoc* test was performed to determine the significance between each pair of participants either by the Wilcoxon rank-sum test for unpaired data or by the Wilcoxon signed-rank test for paired data.

### 2.6. Data/Code availability statement

The data and code used in this study are available from the first and corresponding author upon reasonable request.

## 3. Results

### 3.1. The saccadic latency is shorter in trials with MSS than without MSS in PS but similar in AS and MGS

**FIGURE 2:**
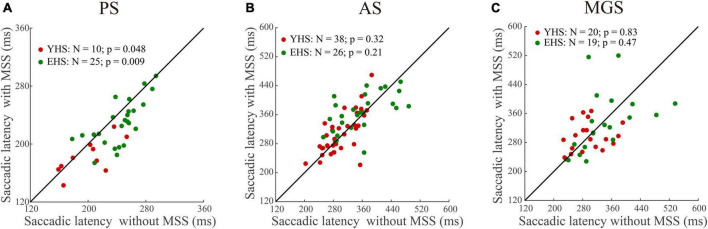
The comparison of saccadic latency between trials with and without MSS. *X*-axis represents the saccadic latency of trials without MSS whereas *Y*-axis represents the saccadic latency of trials with MSS. Red and green circles indicate data of YHS and EHS, respectively. N denotes the number of subjects. **(A–C)** Represents data from pro-saccade task (PS), anti-saccade task (AS), and memory-guided saccade task (MGS), respectively. Saccadic latency of trials with MSS is shorter than that without MSS in PS, whereas saccadic latencies are similar between trials with and without MSS in AS and MGS (Wilcoxon sign-rank tests). YHS, young healthy subjects; EHS, elderly healthy subjects.

To examine whether the automatic theory is universal for the generation of MSS, we compare the saccadic latency between trials with and without MSS in three tasks (Figure 2). Our data show that in PS, the saccadic latency in trials with MSS was significantly shorter than that without MSS in both YHS and EHS (Figure 2A, *p* = 0.048, effect size = 0.3 and *p* = 0.009, effect size = 0.25 for YHS and EHS, Wilcoxon signed-rank test); however, in AS and MGS, the saccadic latency was similar between trials with and without MSS in both YHS and EHS (Figures 2B–C), AS task, *p* = 0.32 and *p* = 0.21; MGS task, *p* = 0.83 and *p* = 0.47 for YHS and EHS, Wilcoxon signed-rank test). Such results indicated that the automatic theory is not applicable for the generation of MSS in AS and MGS.

### 3.2. The ISIs of the MSS are similar among the three tasks

**FIGURE 3:**
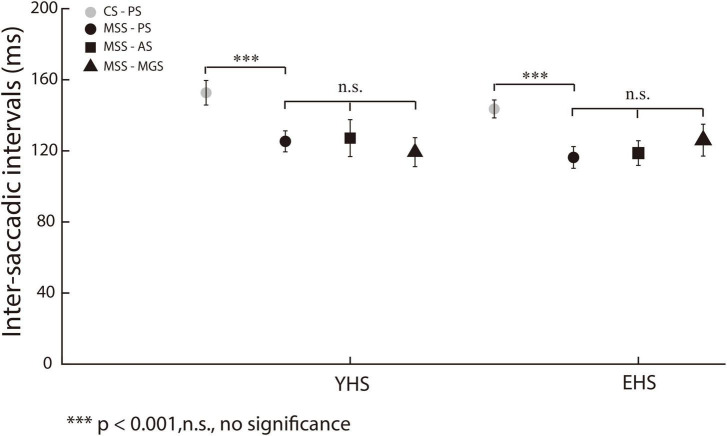
ISIs of MSS and CS among three saccadic tasks. *X*-axis represents YHS and EHS. *Y*-axis represents ISI. The ISIs of MSS and CS were indicated by black and gray color, respectively. The ISIs of CS were significantly higher than that of MSS. Black circle, square and triangle indicate the ISIs of MSS in PS, AS, and MGS, respectively. The ISIs of MSS among three tasks remain similar in both YHS and EHS. Error bars show the standard error of the mean; ^***^denotes *p* < 0.001, n.s. denotes no significant difference (Wilcoxon rank-sum and Kruskal–Wallis test).

The signature of error correction by the forward internal model is to make a comparison between the desired and executed saccades (Kawato, 1999; Mehta and Schaal, 2002). Therefore, we assume that MSS is generated by internal model for saccadic error correction. If such assumption is held, the ISIs of MSS should be shorter than the latency of externally triggered saccades, i.e., single saccades and one primary saccade followed by one CS, because the latter is involved in the process of visuomotor transformation and needs longer time to be completed. The primary/first saccades in the present study are typically visual triggered saccades. Thus, we made comparison between the mean ISIs of MSSs and the mean latency of the first saccades including trials with and without MSS in the same task and same subject. Indeed, our data show that ISIs remain at a similar level (∼120 ms) among PS, AS and MGS in YHS and EHS (Figure 3, black symbols, *p* = 0.15 and *p* = 0.30 for YHS and EHS, Kruskal–Wallis test, corrected by Bonferroni correction). Meanwhile, when comparing the mean ISIs of MSSs with the mean saccadic latency in the same task and subjects, the former was found to be significantly smaller than the latter (Table 2, PS, YHS: *p* = 8.9e-12, effect size = 0.70, EHS: *p* = 1.03e-17 and effect size = 0.92; AS, YHS: *p* = 5.2e-15, effect size = 0.83, EHS: *p* = 2.3e-15, effect size = 0.92; MGS, YHS: *p* = 1.9e-13, effect size = 0.86, EHS: *p* = 1.3e-12, effect size = 0.90).

**TABLE 2:** ISIs of MSS and primary saccadic latency of YHSs and EHSs.

Group	Saccadic task	ISIs of MSS^a^	Saccadic latency^a^	*p*-value^b^
YHS	PS	125.34 ± 36.43	199.14 ± 37.91	<0.0001
	AS	127.17 ± 68.91	307.89 ± 49.23	<0.0001
	MGS	119.27 ± 46.64	283.47 ± 49.09	<0.0001
EHS	PS	116.31 ± 26.12	254.27 ± 29.89	<0.0001
	AS	118.75 ± 42.80	392.94 ± 70.78	<0.0001
	MGS	126.03 ± 49.13	392.26 ± 73.28	<0.0001

^a^Mean ± SD (ms).

^b^*p*-value corresponds to the statistical results between the ISIs of MSS and saccadic latency in each saccadic task and group by Wilcoxon rank-sum test.

ISIs, inter-saccadic intervals; MSS, multiple step saccades; YHS, young healthy subjects; EHS, elderly healthy subjects; PS, pro-saccade task; AS, anti-saccade task; MGS, memory-guided saccade task.

In addition, the small amplitude of saccades in PS have been separated into MSS and CS according to our criterion (see section “Materials and methods” for detailed information). Thus, we also compared the mean ISIs of the MSS with the mean ISIs of the CSs in the same task and same subjects. Our results show that the mean ISIs of MSS were significantly shorter than those of CSs (Figure 3, black and gray circles, *p* = 4.3e-05, effect size = 0.30 and *p* = 5.3e-15, effect size = 0.40 for YHS and EHS, Wilcoxon rank-sum test).

Our results showed that: (1) the mean ISIs of MSS among PS, AS and MGS remained in a similar level; (2) the mean ISIs of MSS were smaller than that of CS; (3) the mean ISI of MSS were smaller than the latency of first/primary saccades. These results support the internal model hypothesis.

### 3.3. The incidences of MSS in voluntary saccades are higher than those in reactive saccades

**FIGURE 4:**
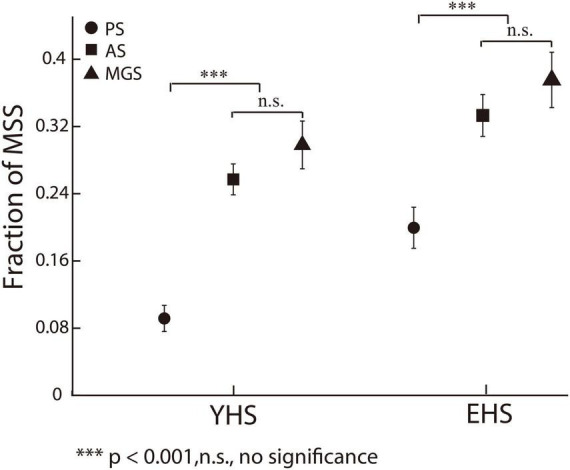
The incidences of MSS among three saccadic tasks. *X*-axis represents YHS and EHS. *Y*-axis represents the fraction of MSS. Black circle, square and triangle indicate the incidences of MSS in PS, AS, and MGS, respectively. The incidences of MSS in AS and MGS are higher than that in PS, whereas there is no significant difference between the incidence of MSS in AS and MGS. Error bars show the standard error of the mean; ^***^denotes *p* < 0.001, n.s. denotes no significant difference (Wilcoxon rank-sum test).

It is well known that subjects make more spatial errors in voluntary saccades (e.g., AS and MGS) than in reactive saccades (e.g., PS) (Munoz et al., 1998; Amador et al., 2006; Gurvich et al., 2007; Blekher et al., 2009; Ka et al., 2016; Mack et al., 2020). One important mechanism is that more oculomotor structures are necessarily involved in the control of voluntary saccades than in reactive saccades (Rottach et al., 1996). When a more complex process is used to produce saccadic commands, more spatial errors will be produced during saccadic execution. According to the working principle of internal models, we assume that there will be more MSS in voluntary saccades than in reactive saccades. To examine this assumption, we directly compared the incidence of MSS in PS with that in AS and MGS. The results show that the incidences of MSS in AS and MGS were significantly higher than those in PS (Figure 4, PS versus AS, *p* = 1.8e-09, effect size = 0.56 and *p* = 1.01e-04, effect size = 0.39 for both YHS and EHS; PS versus MGS, *p* = 1.7e-08, effect size = 0.56 and *p* = 3.8e-05, effect size = 0.44 for both YHS and EHS, Wilcoxon rank-sum test), whereas there was no significant difference between AS and MGS (Figure 4, *p* = 0.33 and *p* = 0.35 for both YHS and EHS, Wilcoxon rank-sum test). Such results provide additional evidence to support our hypothesis that the generation of MSS is due to error correction by internal models.

## 4. Discussion

In the present study, we found that, consistent with previous findings (Van Donkelaar et al., 2007), the saccadic latency in trials with MSS was significantly shorter than that without MSS in PS (Figure 2A). However, in AS and MGS, the saccadic latency was similar between trials with and without MSS (Figures 2B–C). In addition, our data showed that the ISIs of the MSS were approximately 120 ms among the three tasks in the YHS and EHS (Figure 3), which was significantly shorter than the saccadic latency of the primary/first saccade (Table 2) and the ISIs of the CS (Figure 3). Furthermore, our results showed that while the MSS incidences were similar between AS and MGS, they were significantly higher than those in PS (Figure 4). Based on these findings, we conclude that the automatic theory is not universally applicable to the generation of MSSs; alternatively, they are generated by error correction during execution of saccades (e.g., forward internal model).

### 4.1. Automatic theory is not universally applicable for the generation of MSSs

Although it has been noted that the incidence of MSS could be a behavioral biomarker for the diagnosis of neurodegenerative diseases, particularly PD (Blekher et al., 2009; Ma et al., 2022), surprisingly, the mechanisms underlying the generation of MSS have rarely been studied. To the best of our knowledge, only one study argued that the occurrence of MSS was due to the automatic saccadic plan (Van Donkelaar et al., 2007). This argument was based on the observation that trials with MSS had shorter saccadic latency than trials without MSS in a visually guided reactive saccade task (Van Donkelaar et al., 2007). However, whether the automatic theory is universally applicable in the generation of MSSs has never been verified by other saccadic tasks. In the present study, we addressed this question by comparing saccadic latencies between trials with and without MSS in three tasks, including reactive saccades (i.e., PS) and voluntary saccades (i.e., AS and MGS). Our working hypothesis is that if the automatic theory is universally applied to the generation of the MSS, the saccadic latency in trials with the MSS would be shorter than that without the MSS in three tasks. In fact, our results show that in AS and MGS, the saccadic latency is similar between trials with and without MSS (Figures 2B–C). Therefore, our results do not support that the automatic theory is universally applicable for the generation of an MSS.

### 4.2. The error correction by the internal model during the execution of saccades is a possible mechanism for the generation of MSSs

We and others have proposed that the function of MSS shares certain similarities with CS, i.e., to correct the saccadic errors between desired/planned and actual/executed eye displacement (Weber and Daroff, 1972; Troost et al., 1974; Kimmig et al., 2002; Ma and Zhang, 2022). It is well known that CS could be triggered by the spatial error between saccadic endpoints and saccadic targets in prosaccades (Lanc and France, 1971; Cohen and Ross, 1978; Tian et al., 2013) or between saccadic endpoints and desired saccadic goals in AS (Hallett, 1978) and MGS (Fujita et al., 2002; Srimal and Curtis, 2010). Since saccades are toward the visual stimuli in PS, there are both internally generated MSSs and externally generated CSs. In contrast, since saccades are toward the mirror location of visual stimulus in AS and toward the remembered location in MGS, there are only internally generated error corrections, which are defined as MSS in the present study. Thus, to some extent, we assume that the neural mechanisms underpinning MSS and CS generation are varied. Regarding CS, the error signal is generated in the visual system between the fovea and the projecting location of the visual stimulus on the retina (Lanc and France, 1971; Tian et al., 2013). Regarding the MSS, the error signal is generated by the comparison between the desired/planned and actual/executed eye displacements (Weber and Daroff, 1972; Troost et al., 1974; Kimmig et al., 2002; Ma and Zhang, 2022). We argue that, in PS, the error signal of MSS is internally generated and the error signal of CS is externally generated is based on the observations of: (1) the ISIs of MSS among PS, AS, and MGS remained in a similar level; (2) the ISIs of MSS were smaller than that of CS; (3) the mean ISIs of MSS were smaller than the latency of first/primary saccades. Supportively, a line of empirical studies have found that neuronal activity reflects the real-time comparison between desired/planned and actual/executed eye positions in monkeys’ cerebellum (Catz et al., 2008; Prsa et al., 2009), and cerebellar lesions cause prolonged ISIs (Federighi et al., 2011), decreased saccadic gain (Takagi et al., 2003), and impaired saccadic adaptation (Panouillères et al., 2013). In addition, according to the results of our previous studies, we found that the incidence of MSS and CS correlate differently with aging (Ma and Zhang, 2022).

The rationale of the forward internal model requires two internally generated signals, i.e., the desired (intentional) and actual (executional) eye displacement signals, to generate an error signal and trigger the CSs (Robinson, 1973; Kawato, 1999; Mehta and Schaal, 2002; Wolpert et al., 2006). Since these two signals are generated before or during the execution of saccades, we believe that the latency to generate MSSs should be similar regardless of the saccadic tasks and shorter than that of externally triggered saccades. Indeed, our data show that (1) the ISIs of MSSs are similar (∼120 ms) among the three tasks in both YHS and EHS (Figure 3); (2) the ISIs of MSSs are significantly shorter than the latency of CS in PS (Figure 3); and (3) the ISIs of MSS are significantly shorter than the latency of primary/first saccades in the three tasks (Table 2). Such results fit well with our speculation that MSS is generated by a forward internal model for real-time saccadic error correction (Robinson, 1973; Kawato, 1999; Mehta and Schaal, 2002; Wolpert et al., 2006; Ma and Zhang, 2022).

### 4.3. The higher oculomotor structures in cortical and subcortical regions affect the generation of MSS

Our results show that the incidences of MSS in AS and MGS are significantly higher than those in PS (Figure 4), whereas there is no significant difference between AS and MGS (Figure 4). Such results are consistent with previous findings that MSS is more frequently observed in voluntary saccades than in reactive saccades (Kimmig et al., 2002; Blekher et al., 2009). Since the generation of voluntary saccades requires the involvement of more oculomotor structures in cortical and subcortical regions than the generation of reflective saccades (Gaymard et al., 1998; Zhang and Barash, 2000, 2004; Coe and Munoz, 2017), our results suggest that the higher-level oculomotor structures in cortical and subcortical regions affect the generation of MSS. Supportively, previous studies have found that suppressing the activity of the frontal eye field (FEF) and supplementary eye field by transcranial magnetic stimulation (TMS) increased the incidence of MSS (van Donkelaar et al., 2009). Moreover, damage of dopaminergic neurons in the basal ganglia increased the incidence of MSS, as observed in PD patients (Kimmig et al., 2002; Blekher et al., 2009) and in PD monkeys (Tereshchenko et al., 2015).

Regarding how higher oculomotor structures affect the generation of MSS, one possible explanation is that the more complicated process to produce saccadic command (e.g., in the control of voluntary saccades) will add more errors in it. As a result, more CSs are required to correct those saccadic errors so that the incidence of MSS in voluntary saccades is higher than that in reactive saccades.

### 4.4. Limitations of the study

Since the present study is a psychophysical experiment, we did not directly assess the relationship between neural activity and the incidence of MSS. Thus, the proposed mechanism of the forward internal model underlying MSS generation is based on the findings of previous studies on the neural control of saccades. Physiological and functional experiments to directly examine our hypothesis are required in future studies.

## 5. Conclusion

Our results indicate that the automatic theory is not a universal mechanism for the generation of an MSS. Instead, the forward internal model for saccadic error correction might be an important mechanism for the generation of the MSS.

## Data availability statement

The original contributions presented in this study are included in the article/supplementary material, further inquiries can be directed to the corresponding author.

## Ethics statement

This studies was involving human participants were reviewed and approved by Beijing Normal University. The patients/participants provided their written informed consent to participate in this study.

## Author contributions

WM and MZ designed the experimental paradigms. WM performed the experiments, analyzed the data, and wrote the manuscript. MZ supervised the experiments and wrote the manuscript. Both authors contributed to the article and approved the submitted version.

## References

[B1] Amador S. C. Hood A. J. Schiess M. C. Izor R. Sereno A. B. (2006). Dissociating cognitive deficits involved in voluntary eye movement dysfunctions in Parkinson’s disease patients. *Neuropsychologia* 44 1475–1482. 10.1016/j.neuropsychologia.2005.11.015 16376954

[B2] Becker W. Fuchs A. F. (1969). Further properties of the human saccadic system: Eye movements and correction saccades with and without visual fixation points. *Vis. Res.* 9 1247–1258. 10.1016/0042-6989(69)90112-6 5360604

[B3] Blekher T. Weaver M. Rupp J. Nichols W. C. Hui S. L. Gray J. (2009). Multiple step pattern as a biomarker in Parkinson disease. *Park. Relat. Disord.* 15 506–510. 10.1016/j.parkreldis.2009.01.002 19211293 PMC2872983

[B4] Catz N. Dicke P. W. Thier P. (2008). Cerebellar-dependent motor learning is based on pruning a Purkinje cell population response. *PNAS* 105 7309–7314. 10.1073/pnas.0706032105 18477700 PMC2438246

[B5] Coe B. C. Munoz D. P. (2017). Mechanisms of saccade suppression revealed in the anti-saccade task. *Philos. Trans. R. Soc. B Biol. Sci.* 372:20160192. 10.1098/rstb.2016.0192 28242726 PMC5332851

[B6] Cohen M. E. Ross L. E. (1978). Latency and accuracy characteristics of saccades and corrective saccades in children and adults. *J. Exp. Child Psychol.* 26 517–527. 10.1016/0022-0965(78)90130-3 744955

[B7] Corin M. S. Elizan T. S. Bender M. B. (1972). Oculomotor function in patients with Parkinson ’ s disease. *J. Neurol. Sci.* 15 251–265. 10.1016/0022-510X(72)90068-8 5014091

[B8] Faul F. Erdfelder E. Lang A.-G. Buchner A. (2007). G*Power 3: A flexible statistical power analysis program for the social, behavioral, and biomedical sciences. *Behav. Res. Methods* 39 175–191. 10.3758/BF03193146 17695343

[B9] Federighi P. Cevenini G. Dotti M. T. Rosini F. Pretegiani E. Federico A. (2011). Differences in saccade dynamics between spinocerebellar ataxia 2 and late-onset cerebellar ataxias. *Brain* 134 879–891. 10.1093/brain/awr009 21354979

[B10] Fujita M. Amagai A. Minakawa F. Aoki M. (2002). Selective and delay adaptation of human saccades. *Cogn. Brain Res.* 13 41–52. 10.1016/S0926-6410(01)00088-X 11867249

[B11] Gaymard B. Ploner C. J. Rivaud S. Vermersch A. I. Pierrot-Deseilligny C. (1998). Cortical control of saccades. *Exp. Brain Res.* 123 159–163. 10.1007/s002210050557 9835405

[B12] Gurvich C. Georgiou-Karistianis N. Fitzgerald P. B. Millist L. White O. B. (2007). Inhibitory control and spatial working memory in Parkinson’s disease. *Mov. Disord.* 22 1444–1450. 10.1002/mds.21510 17516454

[B13] Hallett P. E. (1978). Primary and secondary saccades to goals defined by instructions. *Vis. Res.* 18 1279–1296. 10.1016/0042-6989(78)90218-3 726270

[B14] Hotson J. R. Langston E. B. Langston J. W. (1986). Saccade responses to dopamine in human MPTP-induced parkinsonism. *Ann. Neurol.* 20 456–463. 10.1002/ana.410200404 3491578

[B15] Jones G. M. DeJong J. D. (1971). Dynamic characteristics of saccadic eye movements in Parkinson’s disease. *Exp. Neurol.* 31 17–31. 10.1016/0014-4886(71)90173-7 5554972

[B16] Ka B. Strallow D. Hening W. Poizner H. Ab S. (2016). Control of voluntary and reflexive saccades in Parkinson ’ s disease. *PubMed Commons. Exp. Brain Res.* 129:10550501. 10.1007/s002210050934 10550501

[B17] Kawato M. (1999). Internal models for motor control and trajectory planning. *Curr. Opin. Neurobiol.* 9 718–727. 10.1016/S0959-4388(99)00028-8 10607637

[B18] Kimmig H. Haußmann K. Mergner T. Lücking C. H. (2002). What is pathological with gaze shift fragmentation in Parkinson’s disease? *J. Neurol.* 249 683–692. 10.1007/s00415-002-0691-7 12111300

[B19] Lanc C. P. R. A. France B. (1971). Corrective saccades?: dependence reafferent signals. *Vis. Res.* 15 465–469. 10.1016/0042-6989(75)90022-x 1129993

[B20] Litvinova A. S. Ratmanova P. O. Evina E. I. Bogdanov R. R. Kunitsyna A. N. Napalkov D. A. (2011). Age related changes in saccadic eye movements in healthy subjects and patients with Parkinson’s disease. *Fiziol Cheloveka* 37 161–167. 10.1134/S0362119711010117 21542316

[B21] Lueck C. J. Tanyeri S. Crawford T. J. Henderson L. Kennard C. (1990). Antisaccades and remembered saccades in Parkinson’s disease. *J. Neurol. Neurosurg. Psychiatry* 53 284–288. 10.1136/jnnp.53.4.284 2341840 PMC1014164

[B22] Lueck C. J. Tanyeri S. Crawford T. J. Henderson L. Kennard C. (1992). Saccadic eye movements in Parkinson’s disease: i. delayed saccades. *Q. J. Exp. Psychol. Sect. A* 45 193–210. 10.1080/14640749208401324 1410555

[B23] Ma W. Li M. Wu J. Zhang Z. (2022). Multiple step saccades in simply reactive saccades could serve as a complementary biomarker for the early diagnosis of Parkinson’s disease. *Front. Aging Neurosci.* 14:912967. 10.3389/fnagi.2022.912967 35966789 PMC9363762

[B24] Ma W. Zhang M. (2022). The effects of age and sex on the incidence of multiple step saccades and corrective saccades. *Front. Aging Neurosci.* 14:963557. 10.3389/fnagi.2022.963557 PMC9490418 36158551

[B25] Mack D. J. Heinzel S. Pilotto A. Stetz L. Lachenmaier S. Gugolz L. (2020). The effect of age and gender on anti-saccade performance: Results from a large cohort of healthy aging individuals. *Eur. J. Neurosci.* 52 4165–4184. 10.1111/ejn.14878 32575168

[B26] Mehta B. Schaal S. (2002). Forward models in visuomotor control. *J. Neurophysiol.* 88 942–953. 10.1152/jn.2002.88.2.942 12163543

[B27] Munoz D. P. Broughton J. R. Goldring J. E. Armstrong I. T. (1998). Age-related performance of human subjects on saccadic eye movement tasks. *Exp. Brain Res.* 121 391–400. 10.1007/s002210050473 9746145

[B28] Oliva G. A. (2001). Drug-induced variations in the probability of occurrence of multiple corrective saccades. *Percept. Mot. Skills* 92 687–690. 10.2466/pms.2001.92.3.687 11453194

[B29] Panouillères M. Alahyane N. Urquizar C. Salemme R. Nighoghossian N. Gaymard B. (2013). Effects of structural and functional cerebellar lesions on sensorimotor adaptation of saccades. *Exp. Brain Res.* 231 1–11. 10.1007/s00221-013-3662-6 23963603

[B30] Prsa M. Dash S. Catz N. Dicke P. W. Thier P. (2009). Characteristics of responses of Golgi cells and mossy fibers to eye saccades and saccadic adaptation recorded from the posterior vermis of the cerebellum. *J. Neurosci.* 29 250–262. 10.1523/JNEUROSCI.4791-08.2009 19129401 PMC6664902

[B31] Robinson D. A. (1973). Models of the saccadic eye movement control system. *Kybernetik* 14 71–83. 10.1007/BF00288906 4206845

[B32] Rottach K. G. Riley D. E. Discenna A. O. Zivotofsky A. Z. John Leigh R. (1996). Dynamic properties of horizontal and vertical eye movements in Parkinsonian syndromes. *Ann. Neurol.* 39 368–377. 10.1002/ana.410390314 8602756

[B33] Srimal R. Curtis C. E. (2010). Secondary adaptation of memory-guided saccades. *Exp. Brain Res.* 206 35–46. 10.1007/s00221-010-2394-0 20803135 PMC3166211

[B34] Takagi M. Tamargo R. Zee D. S. (2003). Effects of lesions of the cerebellar oculomotor vermis on eye movements in primate: Binocular control. *Prog. Brain Res.* 142 19–33. 10.1016/S0079-6123(03)42004-9 12693252

[B35] Teräväinen H. Calne D. B. (1980). Studies of parkinsonian movement: 1. Programming and execution of eye movements. *Acta Neurol. Scand.* 62 137–148. 10.1111/j.1600-0404.1980.tb03015.x 7211164

[B36] Tereshchenko L. V. Anisimov V. N. Shul’govsky V. V. Latanov A. V. (2015). Early changes in saccadic eye movement in Hemiparkinsonian MPTP-treated monkeys. *Perception* 44 1054–1063. 10.1177/0301006615596868 26562919

[B37] Tian J. Ying H. S. Zee D. S. (2013). Revisiting corrective saccades: Role of visual feedback. *Vis. Res.* 89 54–64. 10.1016/j.visres.2013.07.012 23891705 PMC3784029

[B38] Troost B. T. Weber R. B. Daroff R. B. (1974). Hypometric saccades. *Am. J. Ophthalmol.* 78 1002–1005. 10.1016/0002-9394(74)90815-0 4613177

[B39] van Donkelaar P. Lin Y. Hewlett D. (2009). The human frontal oculomotor cortical areas contribute asymmetrically to motor planning in a gap saccade task. *PLoS One* 4:e7278. 10.1371/journal.pone.0007278 19789706 PMC2749336

[B40] Van Donkelaar P. Saavedra S. Woollacott M. (2007). Multiple saccades are more automatic than single saccades. *J. Neurophysiol.* 97 3148–3151. 10.1152/jn.01339.2006 17287433

[B41] Van Gisbergen J. A. M. Duysens J. Henderson L. Kennard C. (1992). Saccadic eye movements in Parkinson’s disease: II. Remembered saccades-towards a unified hypothesis? *Q. J. Exp. Psychol. Sect. A* 45 211–233. 10.1080/14640749208401325 1410556

[B42] Weber R. B. Daroff R. B. (1972). Corrective movements following refixation saccades?: type and control system analysis. *Vis. Res.* 12 467–475. 10.1016/0042-6989(72)90090-9 5021911

[B43] White O. B. Saint-cyr J. A. Tomlinson R. D. Sharpe J. A. (1983). Ocular motor deficits in parkinson’s disease: II. Control of the saccadic and smooth pursuit systems. *Brain* 106 (Pt 3), 571–587. 10.1093/brain/106.3.571 6640270

[B44] Wolpert D. M. Ghahramani Z. Jordan M. (2006). An internal model for sensorimotor integration - Wolpert et al. (1995).pdf. *Science* 24 545–554. 10.1038/nphys2178 7569931

[B45] Zhang M. Barash S. (2000). Neuronal switching of sensorimotor transformations for antisaccades. *Nature* 408 971–975. 10.1038/35050097 11140683

[B46] Zhang M. Barash S. (2004). Persistent LIP activity in memory antisaccades: working memory for a sensorimotor transformation. *J. Neurophysiol.* 91 1424–1441. 10.1152/jn.00504.2003 14523076

